# Soluble Extract from *Moringa oleifera* Leaves with a New Anticancer Activity

**DOI:** 10.1371/journal.pone.0095492

**Published:** 2014-04-18

**Authors:** Il Lae Jung

**Affiliations:** Department of Radiation Biology, Environmental Radiation Research Group, Korea Atomic Energy Research Institute, Daejeon, Korea; University of Windsor, Canada

## Abstract

*Moringa oleifera* has been regarded as a food substance since ancient times and has also been used as a treatment for many diseases. Recently, various therapeutic effects of *M. oleifera* such as antimicrobial, anticancer, anti-inflammatory, antidiabetic, and antioxidant effects have been investigated; however, most of these studies described only simple biological phenomena and their chemical compositions. Due to the increasing attention on natural products, such as those from plants, and the advantages of oral administration of anticancer drugs, soluble extracts from *M. oleifera* leaves (MOL) have been prepared and their potential as new anticancer drug candidates has been assessed in this study. Here, the soluble cold Distilled Water extract (4°C; concentration, 300 µg/mL) from MOL greatly induced apoptosis, inhibited tumor cell growth, and lowered the level of internal reactive oxygen species (ROS) in human lung cancer cells as well as other several types of cancer cells, suggesting that the treatment of cancer cells with MOL significantly reduced cancer cell proliferation and invasion. Moreover, over 90% of the genes tested were unexpectedly downregulated more than 2-fold, while just below 1% of the genes were upregulated more than 2-fold in MOL extract-treated cells, when compared with nontreated cells. Since severe dose-dependent rRNA degradation was observed, the abnormal downregulation of numerous genes was considered to be attributable to abnormal RNA formation caused by treatment with MOL extracts. Additionally, the MOL extract showed greater cytotoxicity for tumor cells than for normal cells, strongly suggesting that it could potentially be an ideal anticancer therapeutic candidate specific to cancer cells. These results suggest the potential therapeutic implications of the soluble extract from MOL in the treatment of various types of cancers.

## Introduction

Various types of plants have been used for several centuries worldwide not only as dietary supplements but also as traditional treatments for many diseases [1], [2], [3]. Indeed, the fact that traditional medicines have been widely used worldwide demonstrates the potential of plants as sources of bioactive compounds, including potential antitumor, antioxidant, antiobesity, and antimicrobial molecules. Among these plants, the widely cultivated *Moringa oleifera* (Moringa or drumstick tree), a rapidly growing perennial tree, was used by the ancient Romans, Greeks, and Egyptians, and has been naturalized from the tropics to the sub-Himalayan regions (e.g., India, Pakistan, Bangladesh, and Afghanistan), Oceania, Latin America, Africa and tropical Asia [4], [5], [6], [7].

For centuries, *M. oleifera* has been used as a traditional medicinal source. Additionally, besides being edible, all the parts of the Moringa tree (e.g., pods, seeds, and leaves) have long been employed for the treatment of many diseases, and therefore, it was called a “miracle vegetable” [6], [8], [9]. Since it is a significant source of fats, proteins, beta-carotene, vitamin C, iron, potassium, and other nutrients [7], [10], the Moringa tree is highly nutritious. For these reasons, some parts of this plant have drawn much attention and have been studied for its various biological activities, including antiatherosclerotic [11], immune-boosting [12], anticardiovascular diseases [13], antiviral [1], [14], [15], [16], antioxidant [2], [17], [18], [19], antimicrobial [18], anti-inflammatory [20] properties and tumor-suppressive effects in skin papillomagenesis, hepatocarcinoma cancer, colon cancer, and myeloma [1], [21], [22], [23].

However, only a few studies have reported the anticancer activity of *M. oleifera* leaves (MOL), and most of them have focused on the evaluation of their efficacy with respect to tumor-suppressive activity, but not on the molecular basis of the tumor-suppressive activity. Additionally, most studies have been conducted using solvent extracts of MOL and not their soluble extracts [1], [17], [18], [21], [24].

Solvent extraction is the most frequently used technique for the isolation of bioactive compounds from plants. Therefore, the recovery of bioactive compounds from *M. oleifera* has been typically accomplished using various solvents, such as methanol and ethanol, as well as hot water and buffers [1], [17], [18], [21], [24]. Nevertheless, the majority of the studies focused on solvent extracts because the efficacy of solvent extraction is higher than simple water extraction. In fact, the buffer extract of *M. oleifera* leaves was much less effective than the solvent extracts for hepatocarcinoma cells [1]. Moreover, solvents can dissolve the many useful organic molecules found in plants, such as phenolic compounds.

In the present study, I prepared a cold water-soluble MOL extract and investigated the possibility as anticancer drugs in different types of human cancer cell lines. Finally, the medical value of a water-soluble MOL extract will be discussed.

## Materials and Methods

### Sample Preparation

Dried leaves of *M. oleifera* cultivated in Chinagmai, Thailand, were purchased from GL Networks Co. Ltd. The dried MOL (150 mg) were suspended in 1 mL of cold water (4°C), vigorously vortexed for 30 s, and refrigerated for 5 min to 24 hours. The suspension was vigorously vortexed again for 1 min at room temperature. The water-insoluble parts of the suspension were removed by centrifuging it twice (12,000 rpm, 10 min each), and the supernatants were collected by membrane filtration (0.2-µm filter). The resulting MOL extracts were lyophilized and stored at −20°C for future analysis. For the experiments, the lyophilized MOL extracts were resuspended into DW at a final concentration of 20 mg/mL of protein.

### Cell Culture

All the cancer cells and African green monkey kidney cell line COS-7 used in this study were obtained from American Type Culture Collection (ATCC, USA) and Korean Cell Type Collection (KCTC, KOREA), respectively. The cells were grown in RPMI-1640 medium (i.e., A549, H23, and H358) and DMEM (i.e., MCF-7, A431, HT1080, and COS-7) (Hyclone Lab, USA) supplemented with 10% fetal bovine serum (FBS; Hyclone Lab) and 1% penicillin-streptomycin. Cells were inoculated at a density of 1×10^5^ cells in a 6-well plate and were maintained at 37°C in a humidified atmosphere containing 95% air and 5% CO_2_.

### Cell Proliferation Assay (MTT Assay)

The viability of cells was analyzed by a cell proliferation assay method using tetrazolium salt (MTT) [25]. Cells were adjusted to 3×10^3^ cells/well and inoculated in 100 µL of appropriate culture medium/well in 96-well plates. After 1-d incubation, the cells were treated with various concentrations of MOL extract (0–400 µg/mL). After another 1- or 2-d incubation, 10 µL of Cell Counting Kit-8 (cat. No. CK04, Dojindo Laboratories, Japan) or WST assay reagent (Daeil Lab Service Co, Korea) was added per well and incubated for an additional 4 h. The absorbance at 450 nm was measured with a microplate reader (Model 680 microplate reader, Bio-Rad Laboratories, USA).

### Flow Cytometric Detection

Cells (1×10^5^) were seeded in a 6-well culture plate for 1 d and treated with the MOL extract. After 2 d, the cells were collected, washed with PBS, and fixed with 70% ethanol at 4°C for 2 h in the dark. Fixed cells were washed twice with PBS and stained with propidium iodide (PI, 50 µg/mL) for 30 min at room temperature. The DNA content was measured with a FACScan system (EPICS XL Flow Cytometry, Beckman Coulter Counter, USA). The percentage of cells in each cell phase was determined using the Phoenix Multicycler Software (Phoenix Flow System).

### Colony-formation Assay

Trypsinized cells were collected and seeded in new 6-well culture dishes at a density of 1×10^3^ cells/well for 1 d before adding the MOL extract. After 7 d, the cells were stained with 0.1% crystal violet and photographed. The experiments were repeated 3 times, and a representative photograph has been provided.

### Measurement of Reactive Oxygen Species (ROS)

Carboxydichlorofluorescein diacetate (DCFH-DA) is a polar compound that is converted into a membrane-impermeable non-fluorescent polar derivative (DCFH) by cellular esterases after its incorporation into the cells. The trapped DCFH is then rapidly oxidized to fluorescent 2′,7′-diclorofluorescein (DCF) by intracellular hydrogen peroxide. Trypsinized cells (approximately 1×10^5^ cells) were washed, resuspended in PBS, and treated with DCFH-DA at a final concentration of 10 µM. The cells were incubated for 30 min in the dark at 37°C, and the ROS level was measured using a FACScan system (EPICS XL Flow Cytometry, Beckman Coulter Counter).

### Microscopy

To monitor cell morphology, cells were visualized by light microscopy (Leica Microsystems, Wetzlar, Germany). Images were captured with a Power Shot S45 Canon Digital Camera system.

### cDNA Synthesis and PCR Amplification

Cells (1 × 10^5^) were seeded in a 6-well culture plate for 1 d before treatment with the MOL extract. After an additional 2-d incubation, total RNA was isolated from the cells by using the High Pure RNA isolation kit (Roche, Basel, Switzerland). To generate first-strand cDNA from the total RNA (1 µg) by using oligo dT, a cDNA synthesis kit (Maxim RT Premix Kit-Oligo dT Primer, iNtRON Biotechnology, Korea) was employed. The resulting cDNAs were amplified with different primers (Table 1) by using Maxim PCR Premix Kit-iTaq (iNtRON Biotechnology, Korea). The amplified polymerase chain reaction (PCR) products were analyzed by 1.5% agarose gel electrophoresis and then photographed under UV light (Smart gel imaging analysis system; Beijing Sage Creation Science And Technology Co. Ltd).

**Table 1:** Primers used in this study.

primer	sequence	PCR condition
Notch1	F: 5'-ATGCTGGAGGACCTCATCAA	55°C-30cycles
	R: 5'-TTGGCCTCCTTGCTTCCACA	
SOX2	F: 5'-CATGAATGCCTTCATGGTGT	54°C-30cycles
	R: 5'-GAGATACATGCTGATCATGTC	
Oct4	F: 5'-CAAAGCTCTGCAGAAAGAACT	55°C-30cycles
	R: 5'-AGCCTCAAAATCCTCTCGTTG	
Klf4	F: 5'-CTGGACCTGGACTTTATTCTCT	54°C-25cycles
	R: 5'-TTCAGCACGAACTTGCCCATCA	
β-actin	F: 5'-CCCAGATCATGTTTGAGACC	55°C-25cycles
	R: 5'-GATGTCCACGTCACACTTCA	
NF-κB	F: 5'-ATAGAAGAGCAGCGTGGGGACT	56°C-30cycles
	R: 5'-GGATGACGTAAAGGGATAGGGC	
c-Myc	F: 5′-AATGAAAAGGCCCCCAAGGTAGTTATCC	55°C-30cycles
	R: 5′-GTCGTTTCCGCAACAAGTCCTCTTC	

### Western Blot Analysis

Cells (1×10^5^) were seeded in a 6-well culture plate for 1 d before treatment with the MOL extract. After an additional 2-d incubation, the cells were collected and lysed for western blot analysis. Antibodies for western blot analysis were purchased from Cell Signaling Technology (β-actin, cat. no. cs4967; Akt, cat. no. cs9272; p-JNK, cat. no. cs9251; p-Erk, cat. no. cs9101; p-IκBα: cat. no. cs2859; SOX2: cat. no. cs3579; and cleaved Notch1, cat. no. cs2421), from Santa Cruz Biotechnology (p53, cat. no. sc-126; cyclinD1, cat. no. sc-753; Notch1, cat. no. sc-6014; NF-κB, cat. no. sc-109; β-catenin, cat. no. sc-796; and c-Myc, cat. no. sc-761), and from Merck Millipore (Oct4, cat. no. mab4305). Protein concentration was determined with the Bradford method (Protein Assay Dye Reagent Concentrate, Bio-Rad Laboratories, cat. no. 500-0006). After cell lysis, equal amounts of proteins (20–80 µg) were separated on a 8–12% SDS polyacrylamide gel according to the size of the proteins and transferred to a nitrocellulose membrane (Whatman). The blots were blocked for 16 h at 4°C with blocking buffer (10% nonfat milk in Tris-buffered saline [TBS] buffer containing 0.1% Tween 20 [TBS-T]). After the membrane was washed with 3 times with TBS-T, it was incubated at room temperature for 2 h with a horseradish peroxidase-labeled secondary antibody and visualized using the ECL kit (GE Healthcare, cat. no. RPN1237). To confirm the transfer of proteins to the nitrocellulose membrane, the membrane was stained with Ponceau S solution (Sigma, cat. no. P7170-1L) for 5 min. The stained membrane was then washed with DW several times, and proper gel transfer was verified.

### Identification of Proteins on SDS-PAGE Gels

Cells (1×10^5^) were seeded in a 6-well culture plate for 1 d before treatment with the MOL extract. The cells were lysed after an additional 2-d incubation. After cell lysis, equal amounts of proteins (20 µg) were separated on a 10% SDS polyacrylamide gel and stained with Comassie blue. The stained gel was destained with a mixed solution of methanol and acetic acid. Bands at around 60–80 kDa on the gel were directly sliced with a knife and treated with trypsin. The tryptic peptides produced by the in-gel digestion were analyzed using an LTQ mass spectrometer (Thermo Finnigan, San Jose, CA) coupled with an Eksigent-Nano-Ultra-UPLC (Eksigent Technologies, CA). Proteins were identified using the UniPort Program (http://www.uniprot.org).

### Microarray Preparation

All the oligonucleotides corresponding to 23,753 coding sequences were resuspended in printing buffer (Telechem International, Inc., USA) at a final concentration of 50 pmol/µL. Resuspended oligonucleotides were spotted onto silanized glass slides (UltraGAPS™, Corning Lifesciences, MA) by using a robotic microarrayer (OmniGrid II, GeneMachines, CA) at 20–25°C with 40% humidity.

### Preparation of the cDNA Probe and Microarray Hybridization

Cells (1×10^5^) were seeded in a 6-well culture plate for 1 d before treatment with the MOL extract. After an additional 2-d incubation, total RNA was extracted. The synthesis of target cDNA probes and hybridization were performed as previously described [26]. Each 20 µg aliquot of total RNA was mixed with 5 µg of pdN6 primer (Amersham Biosciences, UK) in 15.4 µL of RNase-free water and incubated at 65°C for 10 min. cDNA was synthesized in the presence of 3 µL of Cy3- or Cy5-dUTP (1 mM each; NEN Life Science Products, Boston, USA) at 42°C for 2 h. The fluorescent-labeled cDNA was purified using a PCR purification kit (Qiagen). Both Cy3 and Cy5-labeled cDNAs were concentrated into a final volume of 27 µL by using Microcon YM-30 (Millipore Corp. USA). The hybridization mixture (80 µL) contained 20 µL of 20× SSC, 8 µL of 1% SDS, 24 µL formamide (Sigma, USA), 10 µg of salmon sperm DNA (Invitrogen Corp.) and 27 µL of labeled cDNA solution. The hybridization mixture was heated at 100°C for 2–3 min and immediately applied onto microarrays. The arrays were hybridized at 42°C for 12–16 h in a humidified hybridization chamber (Array Chamber X, GenomicTree Inc., Korea). The hybridized microarrays were washed, and quantification was performed using an Axon 4000B scanner (Axon Instruments, CA).

### Data Acquisition and Analysis

The hybridization images were used for quantification with GenePix Pro 4.0 (Axon Instruments, CA). The average fluorescence intensity for each spot was calculated, and the local background was subtracted. All data normalization and statistical analyses were performed using GeneSpring 6.1 (Silicon Genetics, USA). Genes were filtered according to their intensity in the control channel. Intensity-dependent normalization (LOWESS) was performed, where the ratio was reduced to the residual of the Lowess fit of the intensity vs. ratio curve. The fold-change values were calculated by dividing the median of the normalized signal channel intensity by the median of the normalized control channel intensity. The ratios of fold changes were calculated by dividing the value of fold change with the value of fold change between relevant conditions. A value above 2.0 was considered significantly different. The parametric ANOVA test was performed using the Benjamini and Hochberg false discovery rate corrections below *p* values of 0.05 or 0.01 to identify genes differentially expressed across the samples. Hierarchical clustering was performed by similarity measurements based on Pearson correlations around 0. The correlation analysis was performed using Pearson correlation (from −1 to 1). The microarray data from these experiments are available for download at http://218.48.131.23/Servelet/GeNet with a guest login.

## Results

### Antiproliferative Effects of Soluble MOL Extract on Lung Cancer Cell A549

**Figure 1:**
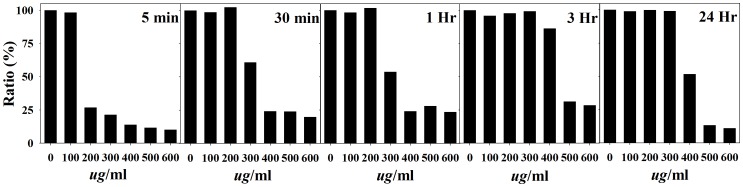
Inhibitory effects of the MOL extract on the proliferation of A549 lung adenocarcinoma cells. The MOL extract was obtained after extraction with cold DW for 5-d incubation. Data were averaged from 3 independent experiments, and the variations were below 10%.

To determine the inhibitory effect on human lung cancer cell A549, various soluble extracts from MOL were prepared using different extraction times. I fixed the temperature at 4°C and investigated the effects of different extraction times (0–24 hours) by MTT analysis. As shown in Figure 1, cell growth inhibition was the highest with the MOL extract obtained after extraction for 5 min.

**Figure 2:**
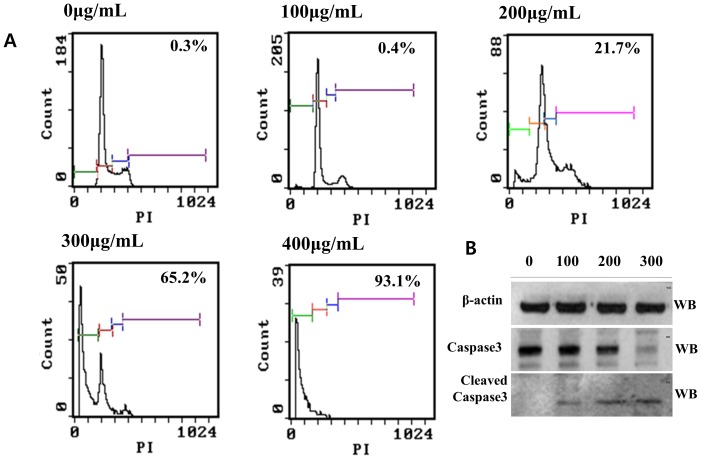
Induction of apoptosis in A549 cells by MOL treatment. (A) FAScan analysis. The numbers in the data boxes indicate the G0/G1 fraction. (B) Western blot analysis of caspase-3. Data were averaged from 3 independent experiments, and the variations were below 10%.

Next, I investigated the changes in the cell cycle and apoptosis in A549 cells. After 48 h-treatment, 200, 300, and 400 µg/mL MOL extracts increased the average sub-G1 population for 6 independent experiments by 21%, 65%, and 93%, respectively (Figure 2A). Western blot analysis showed that caspase-3 was downregulated and that cleaved caspase-3 was upregulated upon MOL treatment in a dose-dependent manner.

**Figure 3:**
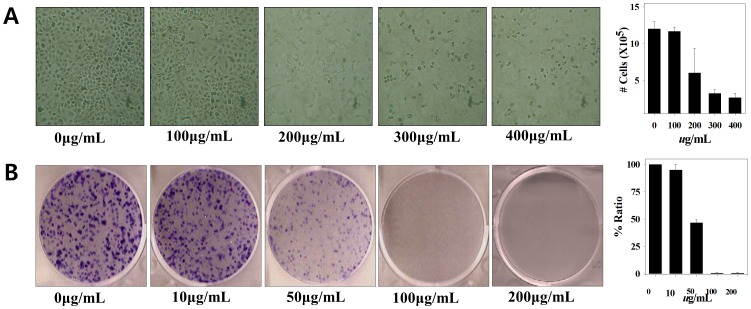
Inhibitory effect of MOL treatment on cell proliferation. (A) Microscopic view (×1,000) and number of cells calculated by the cell counter. (B) Colony-formation assay and number of colonies.

The cytotoxic effect of MOL extract against A549 cells was also examined using microscopy and clonogenic assays. As shown in Figure 3A, cells treated with more than 200 µg/mL of the MOL extract could not adhere onto the culture dishes and showed a typical apoptotic form. The survival ability of A549 cells following treatment with the MOL extracts was assessed using the clonogenic survival assay, in which a total of 3,000 cells were allowed to adhere for 24 h and were subsequently treated with increasing concentrations of MOL extract. After 7 d, the colonies were fixed and stained with crystal violet (0.1% w/v) for 1 min, washed with DW, and photographed. As shown in Figure 3B, the fraction of A549 surviving colonies significantly decreased with increasing amounts of the MOL extract, compared to the results for nontreated cells. No colony was observed above 100 µg/mL concentration.

### Detection of Intracellular ROS

**Figure 4:**
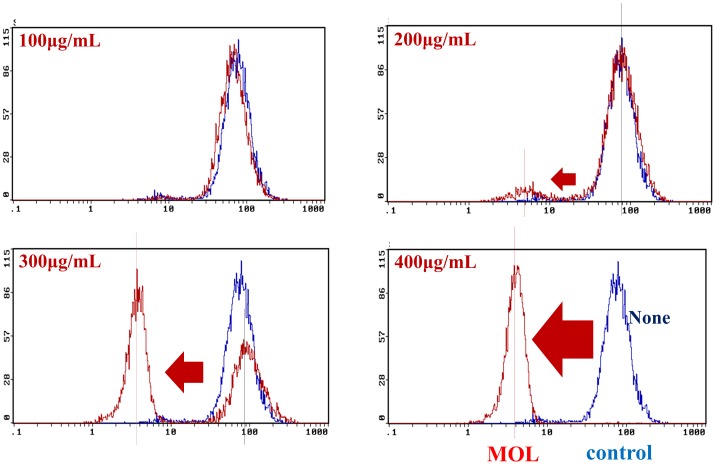
ROS determination by DCFH-DA treatment. Trypsinized cells grown for 48

Next, since the antioxidant effects of MOL extracts have been proven for several cellular and molecular targets associated with cell death and cell survival [27], [28], [29], [30], I measured the intracellular ROS level. A fluorometric assay was used to determine intracellular levels of ROS. The production of ROS following treatment with soluble MOL extracts for 48 h was measured using the cell-permeable oxidation-sensitive dye 2,7-dichlorodihydrofluorescein diacetate (DCFH-DA) (Invitrogen). As shown in Figure 4, the MOL extract induced a significant decrease in ROS concentration as compared to the untreated control group (blue plot) in a dose-dependent manner, indicating that MOL has free radical-scavenging abilities.

### Housekeeping Gene or Protein as a Control

**Figure 5:**
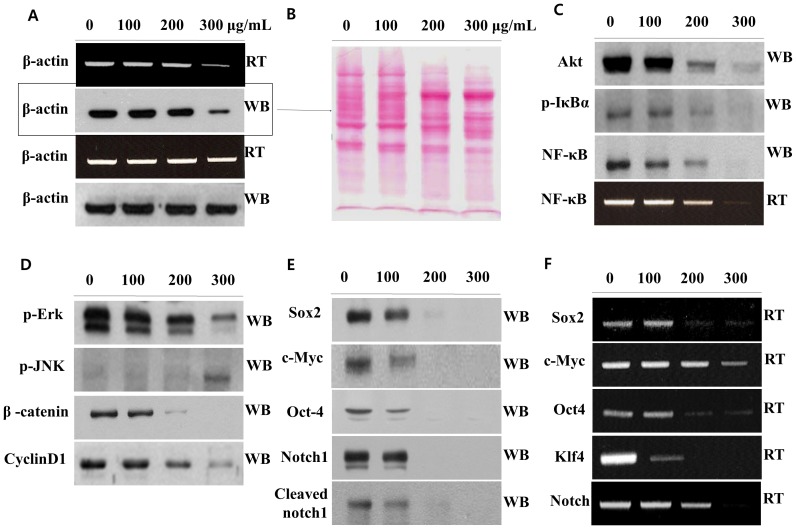
Western blot analysis. Cells were grown for 48(A) β-actin expression. (B) Ponceau S solution staining. (C)–(F) Expression levels of different proteins.

I investigated the expression of apoptosis-related mRNAs and proteins induced by MOL treatment. First, I tested the expression of housekeeping genes or proteins (e.g., β-actin) as a control by reverse transcription (RT)-PCR and western blot analysis, respectively. However, as shown in the first and second gels of Figure 5A, the expression level of β-actin was notably lower in 300 µg/mL MOL-treated cells than in untreated cells, in spite of loading equal amounts of sample on the gels. These phenomena were repeatedly observed in many experiments. In order to confirm that I loaded the same concentration of protein for the western blot analysis, total protein bands were visualized by Ponceau S (Sigma) staining of the membrane, ruling out any problem in gel loading and protein transfer to the membrane (Figure 5B). The reason why abnormal band patterns relative to the 300 µg/mL MOL-treated cells were observed will be discussed below in the context of the next experiments. However, subsequently, I added more RNA and proteins to the 300 µg/mL MOS-treated sample in order to obtain comparable expression levels among all the samples (third and fourth gels in Figure 5A).

### Signaling Pathway

A549 were exposed to 0–300 µg/mL of the soluble MOL extract and mRNA and protein expression was analyzed by RT-PCR and western blot, respectively. Akt, p-IkB, NF-kB, p-Erk, β-catenin, and cyclin D1 were significantly downregulated in MOL-treated cells in a dose-dependent manner (Figure 5C and D). However, c-Jun N-terminal kinases (JNKs), which can be activated by inflammatory signals, changes in levels of ROS, ultraviolet radiation, protein-synthesis inhibitors, and a variety of stresses, were increased by MOL treatment.

Forced expression of Sox, Oct4, Klf4, or c-Myc generates induced pluripotent stem (iPS) cells [31], while silencing of the SOX2 gene reduces the tumorigenic property of A549 cells with attenuated expression of c-MYC, WNT1, WNT2, and NOTCH1 in xenografted NOD/SCID mice [32], [33]. Therefore, I investigated the protein and gene expression of the abovementioned molecules, including that of Notch1 [33] and its active intracellular domain, cleaved Notch1 [34], [35], [36], [37]. Interestingly, as shown in Figure 5E and F, the levels of all the proteins tested showed a significant decrease. Additionally, the dose-dependent decrease in mRNA expression was also consistent with the MOL treatment.

### Significant Decrease in Protein Expression because of MOL Treatment

**Figure 6:**
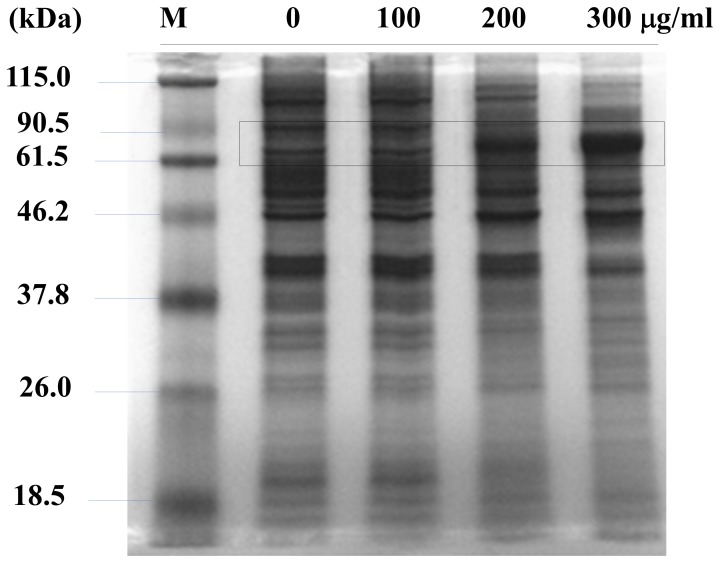
SDS-PAGE analysis for total cellular proteins. Total proteins in cells treated with or without the MOL extract were measured. Equal amounts of total proteins (20 µg) were separated on a 10% SDS polyacrylamide gel, stained with Comassie brilliant blue, and destained. The box highlights the molecular weight region of interest.

As shown in Figure 5A, the expression of the housekeeping protein β-actin was considerably low even if equal amounts of proteins were loaded and normal transfer of proteins to the membrane occurred. For more in-depth analysis, I examined the SDS-PAGE gel patterns. As shown in Figure 6, overall band intensities were similar regardless of the MOL concentrations used, suggesting that relatively similar concentrations of proteins were loaded in each well. However, interestingly, the levels of most of the proteins decreased, with the exception of some proteins in the 61.5–90.5 kDa regions. The main proteins in this range were identified as heat-shock proteins: heat shock cognate 71 kDa protein (69 kDa), heat shock 70 kDa protein (68 kDa), heat shock protein HSP 90-beta (83 kDa) and heat shock protein, mitochondrial (61 kDa).

### Significant Downregulation of Gene Expression by MOL Treatment

**Figure 7:**
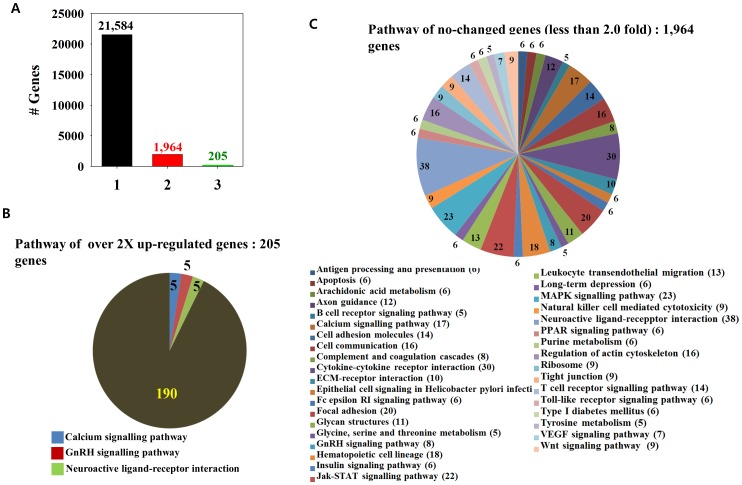
Gene microarray and pathway analyses. (A) Microarray results. (B) Functional analysis related to the most upregulated genes (B) and those showing no change (C).

I showed that MOL led to a significant decrease in the levels of most proteins. Therefore, I investigated total gene expression patterns by the microarray assay (Figure 7A). Of the 23,753 genes tested, only 205 genes (0.9%) were 2-fold upregulated and 21,584 genes (90.1%) of the genes (90.1%) were downregulated. The number of genes that did not show change in expression was 1,964 (8.3%). This microarray result is consistent with the outcome for SDS PAGE, indicating that the significant decrease in protein levels was probably due to the downregulation of mRNA expression. Additionally, the functions of the most upregulated genes have not been elucidated yet (Figure 7B); the functions of the unchanged genes have been shown in Figure 7C.

### Degradation of Ribosomal RNA (rRNA)

**Figure 8:**
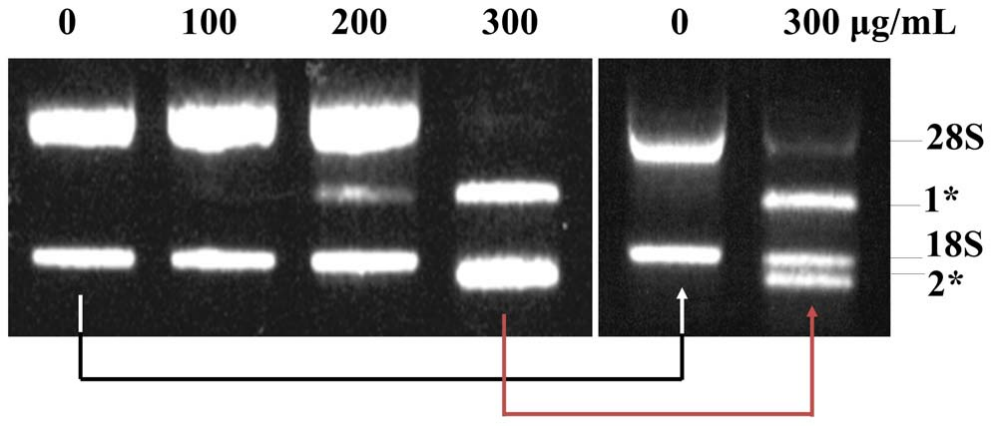
Degradation of rRNA. Normal rRNA shows 2 major bands, 28S and 18S, on the agarose gel. Degraded rRNAs are located between the 28S and 18S rRNA bands (designated as “1”) and below (designated as “2”) the 18S rRNA. In order to investigate the atypical band seen for 300 µg/mL MOL-treated cells in the left panel, more dilute rRNA was separated by gel electrophoresis.

In order to investigate the causes of the significant downregulation of mRNA, I first examined the band pattern of rRNA in the gel after total RNA extraction. The normal pattern of 2 clear 28S and 18S rRNA bands appeared in the range of 0–100 µg/mL MOL; however, slight and significant degradation of rRNA was observed for the 200 µg/mL and 300 µg/mL MOL treatments, respectively (Figure 8, left panel). This dose-dependent rRNA degradation led to the appearance of a new band (designated as “1”) between the 28S and 18S rRNA bands on the gel. Additionally, a new band (designated as “2”) from the 300 µg/mL MOL-treated cells was observed just below the 18S rRNA band. The origin of these new bands needs to be investigated further. The data indicate that significant downregulation of most of the genes resulted from both the decrease in normal RNA patterns and the increase in abnormal RNA patterns.

### Comparison of Cell Viability

**Figure 9:**
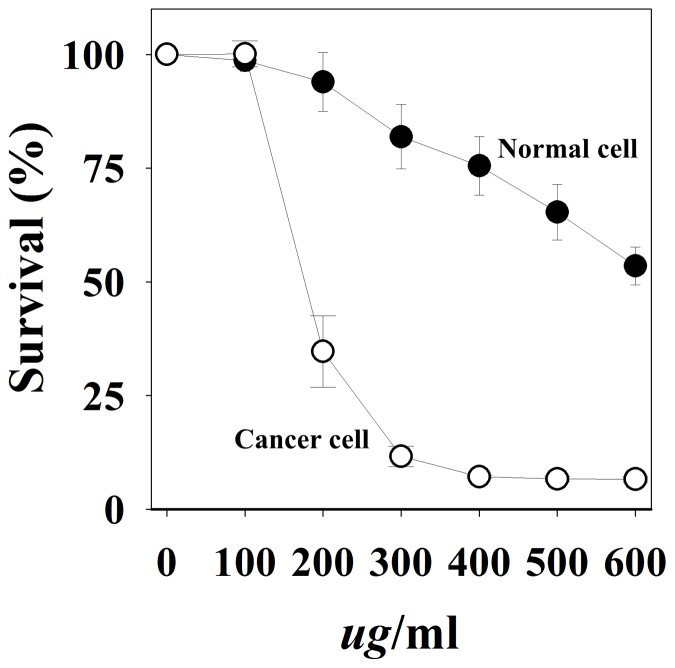
Comparison of cell proliferation between normal and cancer cells. The average values for 3 independent experiments are shown.

The MOL extract was tested for cytotoxic effects against normal cells by using MTT analysis (Figure 9). The analysis showed the considerable toxicity associated with the soluble MOL extract in the cancer cell line, A549 (∼65% for 200 µg/mL). At the same concentration, the viability of the normal cell line (i.e., COS-7) exposed to the MOL extract showed minor cytotoxicity, demonstrating that normal cells are more resistant to the extract than cancer cells. While MOL evoked death of all the A549 cells above 300 µg/mL MOL, normal COS-7 cells showed a gradual decrease in cell viability, with over 50% survival seen even at 600 µg/mL MOL. In conclusion, MOL is highly specific against cancer cells.

## Discussion

Despite the recent advancements in chemotherapeutics, chemotherapy is still associated with severe adverse effects such as nephrotoxicity, nausea, hair loss, skin irritation, anemia, and infertility [38], [39]. Therefore, naturally occurring anticancer compounds from natural plants, especially those with low toxicity and high potency, have important implications for chemotherapy and chemoprevention.

Natural plants have drawn much attention for their pharmacological effects in the treatment and prevention of various diseases due to their high biocompatibility, low toxicity, and potential biological activity [40]. Among them, edible *M. oleifera* is known to be a rich source of various nutrients and has therefore been regarded as an important crop [41]. Additionally, the plant has been considered a multipurpose plant that could be used as a medicinal plant; vegetable; animal fodder; and a source of vegetable oil, which is used in condiments and the manufacture of perfumes, cosmetics, and hair care products [42], [43]. Among the various parts of *M. oleifera*, the roots, pods, seeds, and gum are used to treat rheumatism and to relieve edema and arthritis [5], [44], [45]; the leaves have been reported to have hypocholesterolemic [46], hepatoprotective [1], [47], [48], antimicrobial [49], anti-gastric ulcer [50], antiviral [16], and hypotensive [13] effects and have been used in the prevention of cardiovascular diseases and as antioxidant [2], [11]. However, because of the importance and versatility of the plant, most of the published reports focused on compositional analysis and on its use as a dietary supplement. *M. oleifera* is also used as a health food and cosmetic in many countries, but its medicinal effects have not yet been well established. In particular, only a few studies have been performed on its use as an anticancer drug, and most of them are limited to solvent extracts.

Bioactive compounds from plant materials are typically recovered with different extraction techniques depending on their chemical properties and distribution in the plant [17]. The most frequently used technique for the recovery of active compounds from plants is solvent extraction [17]. Ethanol, methanol, acetone, and ethyl acetate have been widely used to extract bioactive compounds from various plants, including *M. oleifera* leaves [51], [52].

In the field of anticancer drug discovery and development process, compounds with the highest anticancer activities often have bulky hydrophobic groups within their chemical structures, rendering them water insoluble [53]. Low water solubility leads to both formulation issues and serious therapeutic challenges. Administering the poorly soluble drug candidate intravenously might result in serious complications such as embolism and respiratory system failure due to the precipitation of the drug [54], while poor absorption would result from extravascular dosing [55]. Therefore, increasing water solubility and/or developing soluble bioactive compounds with high anticancer activities have attracted increasing attention. In this study, I focused on the new water-soluble MOL extracts and examined its potential as an anticancer drug candidate.

**Figure 10:**
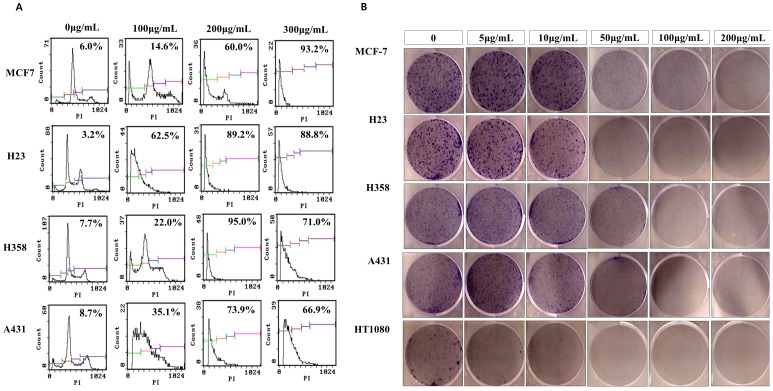
FACS analysis and Inhibitory effect of MOL treatment on the proliferation of different cancer cells. Induction of apoptosis (A) and Colony-formation ability (B) by the MOL extract in different cancer cells was analyzed by FACS and by cologenic assay.

I also demonstrated that concentrations above 300 µg/mL of the cold water (4°C)-soluble MOL extract showed a notable antiproliferative effect in *in vitro* experiments performed using the A549 lung cancer cell line (Figures 1, 2, 3, and 4). Additionally, the MOL extract had an wide spectrum of antiproliferative effect in different cancer cells (Figure 10). As observed by western blot and RT-PCR analysis, many oncogenes and iPS-induction genes were considerably downregulated in A549 cells treated with the extract, demonstrating that the soluble MOL extract can effectively prevent cancer cell proliferation. Although the MOL extract induced severe cell cytotoxicity in A549 cancer cells, however it was not the case anymore in normal cells. As shown in Figure 9, the MOL extract showed less cytotoxicity in normal cells, COS-7, than in cancer cells, A549, demonstrating that normal cells are more resistant to the extract than cancer cells. The reason why the difference in the cell cytotoxicity between cancer cells and normal cells is not clear at this time, but I think complex effects caused by some compounds in the extract can protect normal cells from severe cytotoxicity. Overall, these data suggest that the cold water (4°C)-soluble MOL extract may become a good candidate for anticancer therapy with high specificity and less adverse effects. In conclusion, I demonstrated that the soluble MOL extract may have be a new promising candidate for a natural anticancer drug. Further studies are required in this regard.

Interestingly, more than 99% of the genes in the MOL extract-treated cells did not show changes or were downregulated more than 2 times compared to the control, and only around 1% was upregulated more than 2 times compared to the control (Figure 7). Additionally, protein expression also indicated downregulation (Figure 6). SDS-PAGE gene analysis demonstrated that proteins of the chaperon family were upregulated, indicating that cells treated with MOL had problems in normal translation and were exposed to higher stress levels (Table 2). Because an abnormal rRNA pattern was observed by gel electrophoresis, downregulation of many genes and proteins may occur because of the increase in abnormal RNA through severe RNA degradation (Figure 8). I concluded that the MOL extract induced rRNA degradation, thus showed cell cytoxicity in cancer cells. Several experiments has been designed and conducted to explain the outstanding results of abnormal rRNA degradation, but all the efforts have failed. However, it is evident that the clear bands that the reviewer mentioned were not originated from random cleavage but from the cleavage of the specific site within rRNA or from the appearance of new rRNA, for example, mitochondrial rRNA or unidentified something else. Further studies will be required including sequence analysis of the new bands.

**Table 2:** Upregualted stress proteins by MOL extract.

Accession No.	Description	Gene	MW
E9PKE3	Heat shock cognate 71 kDa protein	HSPA8	69 kDa
F8VZJ4	Heat shock 70 kDa protein	HSPA1B	68 kDa
P08238	Heat shock protein HSP 90-beta	HSP90AB1	83 kDa
P10809	60 kDa heat shock protein, mitochondrial	HSPD1	61 kDa

This table indicates the major bands form Figure 7 located around 60–80 kDa on the gel.

Recently, Tiloke *et al*. [56] have reported that aqueous MOL extract has an antiproliferative effect on cancerous human alveolar epithelial cells. In the study, aqueous MOL extract was prepared “by boiling” the crushed *M. oleifera* leaves. They suggested that the aqueous MOL extract showed approximately 33% inhibition of cell viability in the MOL-treated group compared with the untreated group. Compared to the data, I had much greater inhibition rate of up to 90% by using cold-MOL extract (see Figure 2). The possible difference in anticancer activities between cold- and hot-DW treated MOL extract might be resulted from the heat inactivation of some bioactive molecules within *M. olefeira* leaves, but obvious reason needs to be clarified through further research. In addition, further studies about the anticancer effect among MOL extracts prepared with different temperatures on the cancer cells are also required.

## References

[1] Khalafalla MM, Abdellatef E, Dafalla HM, Nassrallah AA, Aboul-Enein KM, et al (2010) Active principle from *Moringa oleifera* lam leaves effective against two leukemias and a hepatocarcinoma. Afr J Biotech 9: 8467–8471.

[2] Iqbal S, Bhanger MI (2006) Effect of season and production location on antioxidant activity of *Moringa oleifera* leaves grown in Pakistan J Food Compos Anal. 19: 544–555.

[3] Wood M (1997) The book of herbal wisdom: Using plants as medicine: North Atlantic Books press. p.374.

[4] Oliveira JTA, Silveira SB, Vasconcelos KM, Cavada BS, Moreira RA (1999) Compositional and nutritional attributes of seeds from the multiple purpose tree *Moringa oleifera* Lamarck. J Sci Food Agric 79: 815–820.

[5] Fahey JW (2005) *Moringa oleifera*: a review of the medical evidence for its nutritional, therapeutic, and prophylactic properties. Part 1. Trees for Life Journal: a forum on beneficial trees and plants. 1: 5 http://www.TFLJournal.org/article.php/20051201124931586.

[6] Fuglie LJ (1999) The Miracle Tree: Moringa oleifera: Natural Nutrition for the Tropics. Church World Service, Dakar. Revised in 2001 and published as The Miracle Tree: The multiple attributes of Moringa, 68,172.

[7] Mukunzi D, Nsor-Atindana J, Xiaoming Z, Gahungu A, Karangwa E, et al (2011) Comparison of volatile profile of *Moringa oleifera* leaves from Rwanda and China using HS-SPME. Pakistan Journal of Nutrition 10: 602–608.

[8] Faizi S, Siddiqui BS, Saleem R, et al (1995) Fully acetylated carbamate and hypotensive thiocarbamate glycosides from *Moringa oleifera* . Phytochem (Oxford) 38: 957–963. 10.1016/0031-9422(94)00729-d 7766390

[9] Anwar F, Latif S, Ashraf M, Gilani AH (2007) *Moringa oleifera*: A food plant with multiple medicinal uses. Phytother Res 21: 17–25. 17089328 10.1002/ptr.2023

[10] Mahmood KT, Mugal T, Haq IU (2010) *Moringa oleifera*: A natural gift-A review. J Pharmacy 2: 775–781.

[11] Chumark P, Khunawat P, Sanvarinda Y, Phornchirasilp S, Morales NP, et al (2008) The in vitro and ex vivo antioxidant properties, hypolipidaemic and antiatherosclerotic activities of water extract of *Moringa oleifera* Lam leaves. J Ethnopharmacol 116: 439–446. 18249514 10.1016/j.jep.2007.12.010

[12] Miyachi K, Fritzler MJ, Tan EM (2004) Benzyl isothiocyanate inhibits excessive superoxide generation in inflammatory leukocytes: implication for prevention against inflammation-related carcinogenesis. Carcinogenesis 25: 567–575. 14688023 10.1093/carcin/bgh051

[13] Faizi S, Siddiqui B, Saleem R, Saddiqui S, Aftab K (1994) Isolation and structure elucidation of new nitrile and mustard oil glycosides from *Moringa oleifera* and their effect on blood pressure. J Nat Prod 57: 1256–1261. 7798960 10.1021/np50111a011

[14] Waiyaput W, Payungporn S, Issara-Amphorn1 J, Panjaworayan N (2012) Inhibitory effects of crude extracts from some edible Thai plants against replication of hepatitis B virus and human liver cancer cells. BMC Com Alt Med 12: 246–252. 10.1186/1472-6882-12-246 PMC3553072 23216691

[15] Lipipun V, Kurokawa M, Suttisri R, Taweechotipatr P, Pramyothin P, et al (2003) Efficacy of Thai medicinal plant extracts against herpes simplex virus type 1 infection in vitro and in vivo. Antivir Res 60: 175–180. 14638393 10.1016/s0166-3542(03)00152-9

[16] Murakami A, Kitazono Y, Jiwajinda S, Koshimizu K, Ohigashi H (1998) Niaziminin, a thiocarbamate from the leaves of *Moringa oleifera*, holds a strict structural requirement for inhibition of tumor-promoter-induced Epstein-Barr virus activation. Planta Med 64: 319–323. 9619112 10.1055/s-2006-957442

[17] Sultana B, Anwar F, Ashraf M (2009) Effect of extraction solvent/technique on the antioxidant activity of selected medicinal plant extracts. Molecules 14: 2167–2180. 19553890 10.3390/molecules14062167 PMC6254218

[18] Kumar V, Pandey N, Mohan V, Singh RP (2012) Antibacterial and antioxidant activity of extract of *Moringa oleifera* leaves-An in vitro study. Int J Pharm Scis Rev Res 12: 89–94.

[19] Kumar NA, Pari L (2003) Antioxidant action of *Moringa oleifera* lam. (drumstick) against antitubercular drugs induced lipid peroxidation in rats. J Med Food 6: 255–259. 14585192 10.1089/10966200360716670

[20] Kumar Gupta S, Kumar B, Srinivasan BP, Nag TC, Srivastava S, et al. (2012) Retinoprotective Effects of Moringa oleifera Via Antioxidant, Anti-Inflammatory, and Anti-Angiogenic Mechanisms in Streptozotocin-Induced Diabetic Rats. J Ocul Pharmacol Ther. In press. 10.1089/jop.2012.0089 23215831

[21] Budda S, Butryee C, Tuntipopipat S, Rungsipipat A, Wangnaithum S, et al (2011) Suppressive effects of *Moringa oleifera* Lam pod against mouse colon carcinogenesis induced by azoxymethane and dextran sodium sulfate. Asian Pac J Cancer Prev 12: 3221–3228. 22471457

[22] Bharali R, Tabassum J, Azad MR (2003) Chemomodulatory effect of *Moringa oleifera* Lam on hepatic carcinogen metabolizing enzymes, antioxidant parameters and skin papillomagenesis in mice. Asian Pac J Cancer Prev 4: 131–139. 12875626

[23] Brunelli D, Tavecchio M, Falcioni C, et al (2010) The isothiocyanate from glucomoringin inhibits NF-kB and reduces myeloma growth in nude mice in vivo. Biochem Pharmacol 79: 1141–1148. 20006591 10.1016/j.bcp.2009.12.008

[24] Guevara AP, Vargas C, Sakurai H, Fujiwara Y, Hashimoto K, et al (1999) An antitumor promoter from *Moringa oleifera* Lam. Mutat Res 440: 181–188. 10209341 10.1016/s1383-5718(99)00025-x

[25] Hansen MB, Nielsen SE, Berg K (1989) “Re-examination and further development of a precise and rapid dye method for measuring cell growth/cell kill. J Immunol Methods. 119: 203–210. 10.1016/0022-1759(89)90397-9 2470825

[26] Tani TH, Khodursky A, Blumenthal RM, Brown PO, Mathews RG (2002) Adaptation to famine: a family of stationary-phase genes revealed by microarray analysis. Proc Natl Acad Sci USA 99: 13471–13476. 12374860 10.1073/pnas.212510999 PMC129697

[27] Lotito SB, Fraga CG (2000) Catechins delay lipid oxidation and alpha-tocopherol and beta-carotene depletion following ascorbate depletion in human plasma. Proc Soc Exp Biol Med 225: 32–38. 10998196 10.1046/j.1525-1373.2000.22504.x

[28] Velayutham P, Babu A, Liu D (2008) Green tea catechins and cardiovascular health: An update. Curr Med Chem 15: 1840–1850. 18691042 10.2174/092986708785132979 PMC2748751

[29] Thangapazham RL, Passi N, Maheshwari RK (2007) Green tea polyphenol and epigallocatechin gallate induce apoptosis and inhibit invasion in human breast cancer cells. Cancer Biol Ther 6: 1938–1943. 18059161 10.4161/cbt.6.12.4974

[30] Kampa M, Alexaki V-I, Notas G (2004) Antiproliferative and apoptotic effects of selective phenolic acids on T47D human breast cancer cells: Potential mechanisms of action breast. Cancer Res 6: R63–R74. 10.1186/bcr752 PMC400651 14979919

[31] Welstead GG, Brambrink T, Jaenisch R (2008) Generating iPS cells from MEFS through forced expression of Sox-2, Oct-4, c-Myc, and Klf4. J Vis Exp 14: 734–735. 10.3791/734 PMC2582849 19066578

[32] Chen S, Xu Y, Chen Y, Li X, Mou W, et al (2012) SOX2 gene regulates the transcriptional network of oncogenes and affects tumorigenesis of human lung cancer cells. PLoS One 7(5): e36326. 22615765 10.1371/journal.pone.0036326 PMC3352903

[33] Lai EC (2004) Notch signaling: control of cell communication and cell fate. Development 131: 965–973. 14973298 10.1242/dev.01074

[34] D’Souza B, Miyamoto A, Weinmaster G (2008) The many facets of Notch ligands. Oncogene 27: 5148–5167. 18758484 10.1038/onc.2008.229 PMC2791526

[35] Katoh M (2007) Notch signaling in gastrointestinal tract (review) Int J Oncol. 30: 247–251. 17143535

[36] Miele L (2006) Notch signaling. Clin Cancer Res 12: 1074–1079. 16489059 10.1158/1078-0432.CCR-05-2570

[37] van Es JH, van Gijn ME, Riccio O, van den Born M, Vooijs M, et al (2005) Notch/gamma-secretase inhibition turns proliferative cells in intestinal crypts and adenomas into goblet cells. Nature 435: 959–963. 15959515 10.1038/nature03659

[38] Khan HA, Alhomida AS (2011) A review of the logistic role of l-carnitine in the management of radiation toxicity and radiotherapy side effects. J Appl Toxicol 31: 707–713. 21818761 10.1002/jat.1716

[39] Sánchez-González PD, López-Hernández FJ, López-Novoa JM, Morales AI (2011) An integrative view of the pathophysiological events leading to cisplatin nephrotoxicity. Crit Rev Toxicol 10: 803–821. 10.3109/10408444.2011.602662 21838551

[40] Li H, Wu WK, Li ZJ, Chan KM, Wong CC, et al (2010) 2,3′,4,4′,5′-Pentamethoxy-trans-stilbene, a resveratrol derivative, inhibits colitis-associated colorectal carcinogenesis in mice. Br J Pharmacol 160: 1352–1361. 20590626 10.1111/j.1476-5381.2010.00785.x PMC2938807

[41] Dahot MU, Memon AR (1985) Nutritive significance of oil extracted from *Moringa oleifera* seeds. J Pharm Univ Karachi 3: 75–80.

[42] Tsaknis J, Lalas V, Gergis V, Douroglou V, Spiliotis V (1999) Characterization of *Moringa oleifera* variety Mbololo seed oil of Kenya. J Agric Food Chem 47: 4495–4499. 10552840 10.1021/jf9904214

[43] Oliveira JTA, Silveira SB, Vasconcelos IM, Cavada BS, Moriera RA (1999) Compositional and nutritional attributes of seeds from the multiple purpose tree *Moringa oleifera* Lamark. J Sci Food Agric 79: 815–820.

[44] Caceres A, Saravia A, Rizzo S, Zabala L, De Leon E, et al (1992) Pharmacologic properties of *Moringa oleifera*. 2: screening for antispasmodic, anti-inflammatory and diuretic activity. J Ethnopharmacol 36: 233–237. 1434682 10.1016/0378-8741(92)90049-w

[45] Mahajan SG, Mali RG, Mehta AA (2007) Protective effect of ethanolic extract of seeds of *Moringa oleifera* Lam. against inflammation associated with development of arthritis in rats. J Immunotoxicol 4: 39–47. 18958711 10.1080/15476910601115184

[46] Ghasi S, Nwobodo E, Ofili JO (2000) Hypocholesterolemic effect of crude leaf of *Moringa oleifera* in high fat diet fed Wister rats. J Ethnopharmacol 69: 21–25. 10661880 10.1016/s0378-8741(99)00106-3

[47] Pari L, Kumar NA (2002) Hepatoprotective activity of *Moringa oleifera* on anti tubercular drug induced liver damage in rats. J Med Food 5: 171–177. 12495589 10.1089/10966200260398206

[48] Fakurazi S, Hairuszah I, Nanthini U (2008) *Moringa oleifera* Lam prevents acetaminophen induced liver injury through restoration of glutathione level. Food Chem Toxicol 46: 2611–2615. 18514995 10.1016/j.fct.2008.04.018

[49] Caceres AB, Cabrera O, Mirals O, Mollinedo O, Imendia A (1991) Preliminary screening for antimicrobial activity of *Moringa oleifera* . J Ethnopharmacol 33: 213–216. 1921416 10.1016/0378-8741(91)90078-r

[50] Dahiru D, Onubiyi JA, Umaru HA (2006) Phytochemical screening and antiulcerogenic effect of *Moringa oleifera* aqueous leaf extract. African J Trad Complement Alternat Med 3: 70–75.

[51] Peschel W, Sanchez-Rabaneda F, Dn W, Plescher A, Gartzia I, Jimenez D, et al (2006) An industrial approach in the search of natural antioxidants from vegetable and fruit wastes. Food Chem 97: 137–150.

[52] Siddhuraju P, Becker K (2003) Antioxidant properties of various extracts of total phenolic constituents from three different agroclimatic origins of drumstick tree (*Moringa oleifera* lam.) leaves. J Agric Food Chem 51: 2144–2155. 12670148 10.1021/jf020444+

[53] Lipinski CA (2000) Drug-like properties and the causes of poor solubility and poor permeability. J Pharmacol Toxicol Methods 44: 235–249. 11274893 10.1016/s1056-8719(00)00107-6

[54] Teicher BA, Andrews PA (2004) Anticancer Drug Development Guide: Preclinical Screening, Clinical Trials and Approval. 2nd ed. Totowa, NJ: Humana Press.

[55] Lipinski CA, Lombardo F, Dominy BW, Feeney PJ (2001) Experimental and computational approaches to estimate solubility and permeability in drug discovery and development settings. Adv Drug Deliv Rev 46: 3–26. 11259830 10.1016/s0169-409x(00)00129-0

[56] Tiloke C, phulukdaree A, Chuturgoon AA (2013) The antiproliferative effect of MOringa oleifera crude aqueous extract on cancerous human alvelor epithelial cells. BMC Complement Altern Med 13: 226–233. 24041017 10.1186/1472-6882-13-226 PMC3852616

